# The peroxisome biogenesis factors posttranslationally target reticulon homology domain-containing proteins to the endoplasmic reticulum membrane

**DOI:** 10.1038/s41598-018-20797-0

**Published:** 2018-02-02

**Authors:** Yasunori Yamamoto, Toshiaki Sakisaka

**Affiliations:** Division of Membrane Dynamics, Department of Physiology and Cell Biology, Kobe University Graduate School of Medicine, Kobe, 650–0017 Japan

## Abstract

The endoplasmic reticulum (ER) is shaped by a class of membrane proteins containing reticulon homology domain (RHD), the conserved hydrophobic domain encompassing two short hairpin transmembrane domains. RHD resides in the outer leaflet of the ER membrane, generating high-curvature ER membrane. While most of the membrane proteins destined to enter the secretory pathway are cotranslationally targeted and inserted into ER membrane, the molecular mechanism how the RHD-containing proteins are targeted and inserted into the ER membrane remains to be clarified. Here we show that RHD-containing proteins can be posttranslationally targeted to the ER membrane. PEX19, a cytosolic peroxin, selectively recognizes the nascent RHD-containing proteins and mediates their posttranslational targeting in cooperation with PEX3, a membrane peroxin. Thus, these peroxisome biogenesis factors provide an alternative posttranslational route for membrane insertion of the RHD-containing proteins, implying that ER membrane shaping and peroxisome biogenesis may be coordinated by the posttranslational membrane insertion.

## Introduction

In eukaryotic cells, diverse types of membrane proteins destined to enter the secretory pathway are inserted into the endoplasmic reticulum (ER) membrane, the starting point of secretion^1,2^. It is widely accepted that the most prominent mechanism of this membrane insertion is the cotranslational pathway mediated by the cytosolic signal recognition particle (SRP), the ER-localized SRP receptor, and the ER-localized translocon consisting of Sec61 complex^3,4^. SRP awaits emergence of the hydrophobic N-terminal signal sequence or the transmembrane domain (TMD) of a membrane protein from the ribosomal exit tunnel and captures it, which in turn pauses the translational elongation of the membrane protein^5,6^. The SRP receptor recruits the SRP-ribosome-nascent polypeptide chain complex to the ER membrane and hands over it to the Sec61 translocon, a protein-conducting channel, followed by the cotranslational insertion of the membrane protein into the ER membrane^7–11^.

While most of the membrane proteins take this SRP-mediated cotralslational route, evidence is accumulating that some classes of the membrane proteins take other posttranslational routes. Tail-anchored (TA) proteins are a class of membrane proteins anchored to the lipid bilayer by a single C-terminal TMD, which encodes the ER targeting information^12,13^. Since the TMD is located very close to their C-termini, the TMD is still inside the ribosome tunnel when the ribosomes terminate the translation of TA proteins, precluding the SRP-mediated cotranslational route^2,14–18^. Therefore, TA proteins have been shown to employ a unique machinery called the TRC40 pathway in mammals or the GET (Guided Entry of TA proteins) pathway in yeasts^2,16–18^ for the posttranslational membrane insertion. After released from the ribosome, the TMD of a nascent TA protein is recognized by TRC40, a cytosolic ATPase^19^, and delivered to the ER membrane^19–21^. CAML and WRB, the two distinct ER membrane proteins, constitute the TRC40 receptor, receive the nascent TA protein from TRC40, and mediate the posttranslational insertion into the ER membrane^22–25^. An alternative backup pathway for TA proteins, called SND (SRP independent-targeting) pathway, has been recently identified in yeast^26^. The SND pathway is composed of one ribosome-associated protein called Snd1 and two ER membrane proteins called Snd2 and Snd3, both of which form a complex with the Sec61 translocon. In agreement with the findings in yeast, hSND2 (also known as TMEM208), a mammalian homolog of Snd2, has been shown to associate with the Sec61 translocon^27^ and mediate insertion of TA proteins into the ER membrane^27,28^, indicating that the SND pathway is evolutionarily  conserved. A recent study has shown that UBXD8, a lipid droplet-destined membrane protein, takes another posttranslational route^29^. Nascent UBXD8 is captured by farnesylated PEX19^29^, a cytosolic peroxisome biogenesis factor (also known as peroxin)^30–32^. PEX19 posttranslatinally targets and inserts UBXD8 into the ER membrane in cooperation with PEX3^29^, an ER-localized peroxisome biogenesis factor that functions as a PEX19 receptor^30–32^, followed by localization of UBXD8 on the lipid droplet. UBXD8 has a single short hairpin TMD encoding the ER-targeting information in the N-terminus and inserts the short hairpin TMD into the outer leaflet of the ER membrane^33,34^. Based on these findings, the previous study has suggested that PEX19 and PEX3 provide the posttranslational route for the lipid droplet-destined membrane proteins having the single short hairpin TMDs^29^, although it has been never demonstrated that the lipid droplet-destined membrane proteins other than UBXD8 indeed take the posttranslational route.

In addition to the TA proteins and UBXD8, there is the possibility that membrane proteins having TMDs responsible for SRP binding might also take a posttranslational route(s) in certain instances. Given that ribosomes translate mRNAs at high speed in mammalian cells (~6 amino acids per second)^35,36^, it is speculated that the high translation speed may cause SRP to sometimes fail to cotranslationally target the membrane proteins whose TMDs encoding the ER targeting information are located near the C-termini. This is also the case with secretory proteins and it has indeed been demonstrated that short secretory proteins whose N-terminal signal sequences encoding the ER targeting information are close to the C-termini can escape from SRP recognition and be posttranslationally targeted and inserted into the ER membrane by calmodulin^37^. Therefore, there may be an as-yet unknown posttranslational route(s) for the membrane proteins escaping from SRP recognition as a backup route. Of note, reticulons, the ER membrane-shaping proteins^38–40^, are promising clients for this possible backup route, because their TMDs encoding the ER targeting information are located near the C-termini as described below. However, it still remains unknown whether reticulons can be posttranslationally targeted and inserted into the ER membrane.

Reticulons are a conserved class of ER membrane-shaping proteins^38–40^ and comprise a family composed of four members, reticulon 1, reticulon 2, reticulon 3 and reticulon 4^41^. Each family member has several splicing isoforms^41,42^. The primary structures of reticulons are characterized by reticulon homology domain (RHD) which encompasses two short hairpin TMDs^38,41,43^. The two short hairpin TMDs reside in the outer leaflet of the ER membrane, which in turn expands the area of the outer leaflet relative to the inner leaflet, generating high curvature membrane such as ER tubules and the edges of ER sheets^38–40,44^. The RHD is not limited to the reticulon family members. We have shown that Arl6IP1 is an ER membrane protein containing the RHD and shapes the ER membrane in the same manner as reticulons^45^. FAM134B, a recently identified receptor for ER-phagy, also contains the RHD and deforms the ER membrane toward the ER-phagy^46^. The mutations in the human genes encoding reticulon 2, Arl6IP1 and FAM134B cause hereditary spastic paraplegia and hereditary sensory neuropathy^47–49^, emphasizing the biological importance of the RHD-containing proteins. The previous study demonstrated that reticulon 3 was cotranslationally targeted to the ER membrane^50^. However, given that the RHD is usually located not far from the C-termini of the proteins, the RHD-containing proteins might sometimes escape from the SRP-mediated cotranslational targeting and take an alternative posttranslational route for membrane insertion as presumed above. In this study, we show that RHD-containing proteins can be posttranslationally targeted to the ER membrane. PEX19 selectively recognizes nascent RHD-containing proteins and mediates the posttranslational targeting in cooperation with PEX3, suggesting that PEX19 and PEX3 constitute an alternative membrane insertion machinery for the RHD-containing proteins.

## Results

### Posttranslational targeting of the RHD-containing proteins to the ER membrane

**Figure 1:**
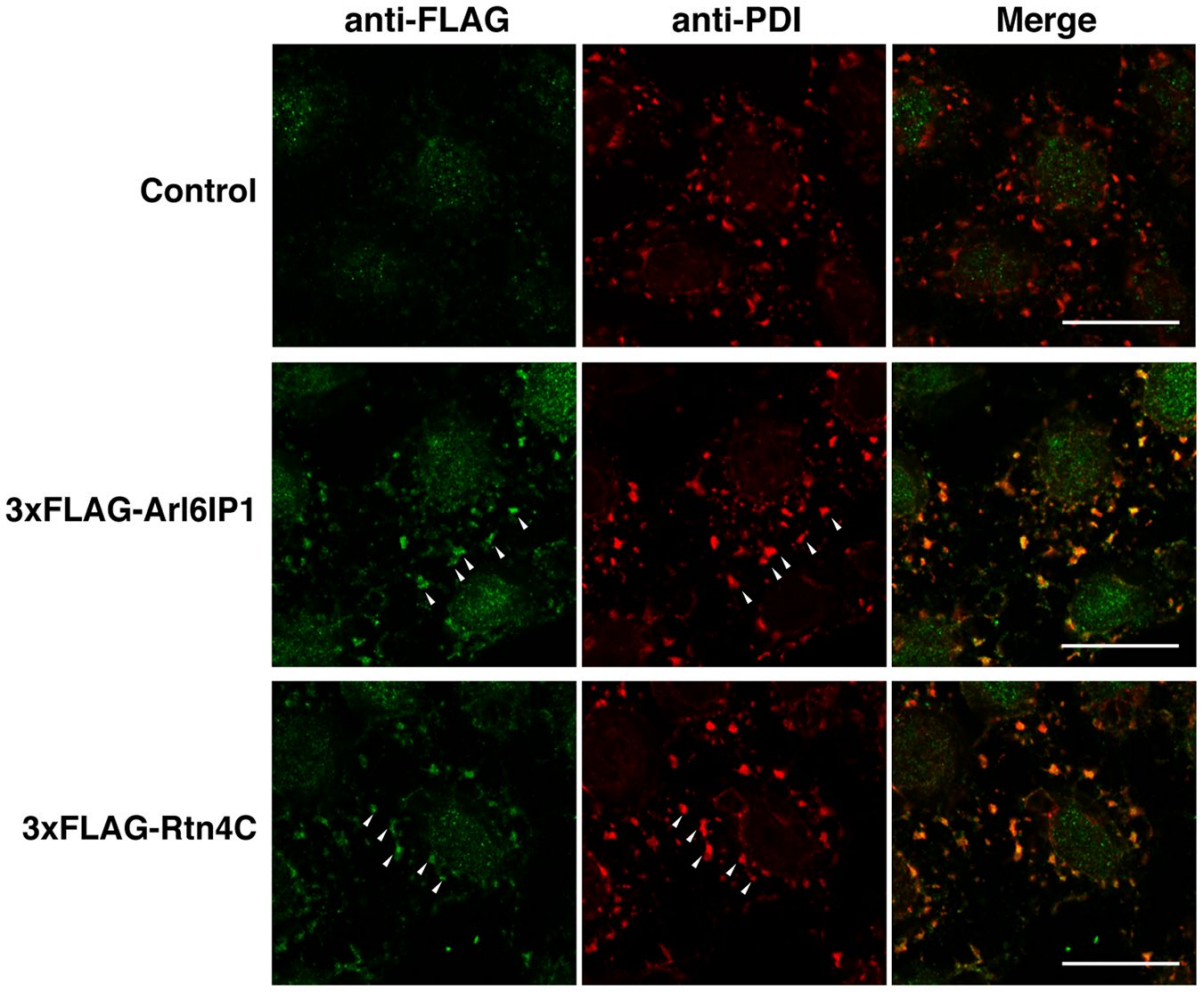
Posttranslational targeting of the RHD-containing proteins to the ER membrane. The rabbit reticulocyte lysate synthesizing 3xFLAG-Arl6IP1 or 3xFLAG-Rtn4C or the rabbit reticulocyte lysate without the 3xFLAG-tagged proteins was supplemented with 1 mM cycloheximide and incubated with the semi-permeabilized HeLa cells, followed by immunostaining with the anti-FLAG pAb and the anti-PDI mAb. Arrowheads indicate the posttranslational targeting to the ER membrane as judged by colocalization of 3xFLAG-Arl6IP1 or 3xFLAG-Rtn4C with PDI. Bars, 20 µm.

To examine whether the RHD-containing proteins were posttranslationally targeted to the ER membrane, we employed an *in vitro* assay using semi-intact cells. HeLa cells grown on coverslips were treated with digitonin to allow selective permeabilization of the plasma membranes. Arl6IP1 and reticulons were chosen as representatives of the RHD-containing proteins. 3xFLAG-tagged Arl6IP1 (3xFLAG-Arl6IP1) and reticulon 4 C (3xFLAG-Rtn4C) were synthesized *in vitro* with rabbit reticulocyte lysates. After the translation reactions were terminated by cycloheximide, the reticulocyte lysate synthesizing 3xFLAG-Arl6IP1 or 3xFLAG-Rtn4C was incubated with the semi-permeabilized HeLa cells, followed by immunostaining with the antibodies against the FLAG tag and protein disulfide isomerase (PDI), an ER marker protein. Compared with the reticulocyte lysate without the 3xFLAG-tagged proteins, strong immunoreactive signals for the FLAG tag were detected in the reticulocyte lysates synthesizing 3xFLAG-Arl6IP1 and 3xFLAG-Rtn4C (Fig. 1). Importantly, the immunoreactive signals for the FLAG tag were well merged with those for PDI. These results indicate that Arl6IP and Rtn4C are posttranslationally targeted to the ER membrane.

We next assessed whether the RHD-containing proteins were posttranslationally inserted into the ER membrane in our *in vitro* assay. The rabbit reticulocyte lysate synthesizing 3xFLAG-Arl6IP1 or 3xFLAG-Rtn4C was incubated with the semi-permeabilized HeLa cells in the presence of cycloheximide to allow posttranslational targeting to the ER membrane in the same manner as described above. After being washed with buffer, the samples were incubated with 0.1 M sodium carbonate (pH11.0) to extract the peripheral membrane proteins, followed by immunostaining with the anti-FLAG pAb and the anti-PDI mAb. Although extraction of the peripheral membrane proteins severely damaged the ER morphology, significant immunoreactivities for 3xFLAG-Arl6IP1 and 3xFLAG-Rtn4C were readily detected at the ER membrane as judged by immunoreactivity for residual PDI (Fig. S1A). Furthermore, we biochemically assessed whether 3xFLAG-Arl6IP1 and 3xFLAG-Rtn4C were resistant to sodium carbonate extraction. HeLa cells were semi-permeabilized with digitonin and suspended in buffer. The rabbit reticulocyte lysate synthesizing 3xFLAG-Arl6IP1 or 3xFLAG-Rtn4C was incubated with the semi-peremabilized HeLa cell suspension in the presence of cycloheximide in a microcentrifuge tube. After being washed with buffer, the semi-peremabilized HeLa cells were suspended in 0.1 M sodium carbonate (pH11.0) and subjected to ultracentrifugation, followed by Western blotting with the anti-FLAG pAb and the anti-PDI mAb. Consistent with the previous study showing that sodium carbonate extracted the peripheral membrane proteins as well as the luminal proteins^51^, more than half of PDI was detected in the supernatants (Fig. S1B). By contrast, almost all of 3xFLAG-Arl6IP1 and 3xFLAG-Rtn4C were detected in the pellets, indicating that 3xFLAG-Arl6IP1 and 3xFLAG-Rtn4C were resistant to sodium carbonate extraction (Fig. S1B). These results suggest that the RHD-containing proteins were not peripherally associated with the ER membrane but posttranslationally inserted into the ER membrane.

### Identification of PEX19 as a protein that selectively binds to the nascent RHD-containing proteins

**Figure 2:**
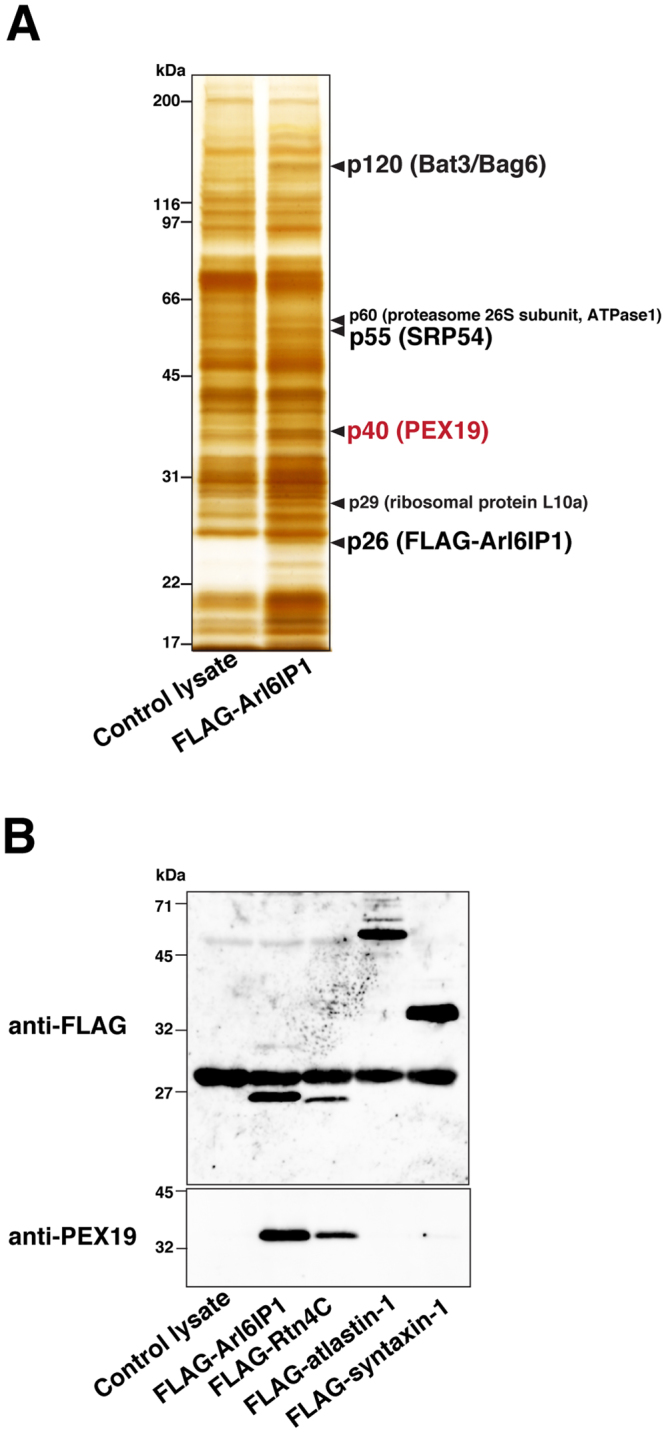
Identification of PEX19 as a protein that selectively binds to the nascent RHD-containing proteins. (**A**) Purification of Arl6IP1-binding proteins from the rabbit reticulocyte lysates. The rabbit reticulocyte lysate synthesizing FLAG-Arl6IP1 was incubated with the anti-FLAG mAb-immobilized agarose. To check the contamination, the rabbit reticulocyte lysate without synthesis of FLAG-Arl6IP1 (control lysate) was also incubated with the anti-FLAG mAb-immobilized agarose. The bound proteins were eluted by competition with a large excess of free FLAG peptide and subjected to SDS-PAGE followed by silver staining. p120, p60, p55, p40 and p29 were identified as Bat3 (also known as Bag6), proteasome 26 S subunit ATPase1, SRP54, PEX19 and ribosomal protein L10a, respectively, by mass spectrometry. (**B**) Selective binding of PEX19 to the nascent RHD-containing proteins. The rabbit reticulocyte lysates synthesizing FLAG-Arl6IP1, FLAG-Rtn4C, FLAG-atlastin-1 and FLAG-syntaxin-1 were supplemented with 1 mM puromycin, followed by incubation with anti-FLAG mAb-immobilized agarose. The bound proteins were eluted with the SDS sample buffer with boiling and subjected to SDS-PAGE followed by Western blotting with the anti-PEX19 pAb and the anti-FLAG pAb.

The results shown in Fig. 1 suggest that the rabbit reticulocyte lysates contain a factor(s) that mediates the posttranslational targeting of the RHD-containing proteins. Therefore, we next sought to purify and identify the targeting factor(s). The rabbit reticulocyte lysate synthesizing FLAG-tagged Arl6IP1 (FLAG-Arl6IP1) was incubated with the anti-FLAG mAb-immobilized agarose. To check the contamination, the rabbit reticulocyte lysate without synthesis of FLAG-Arl6IP1 was also incubated with the anti-FLAG mAb-immobilized agarose. The bound proteins were subjected to SDS-PAGE followed by silver staining. In addition to FLAG-Arl6IP1, five prominent bands at 120, 60, 55, 40 and 29 kDa (p120, p60, p55, p40 and p29) were specifically detected in the eluate from the rabbit reticulocyte lysate synthesizing FLAG-Arl6IP1 (Fig. 2A), and subjected to mass spectrometry analysis. p55 was identified as SRP54, a component of SRP that plays the central role in binding to the signal sequence or the TMD in the cotranslational targeting^52^. This result suggests that, in agreement with the previous report showing the cotranslational targeting of Rtn3^50^, Arl6IP1 can be cotranslationally targeted by the SRP and inserted into the ER membrane presumably through the translocon. p120, p60, p40 and p29 were identified as Bat3 (also known as Bag6)^53–55^, proteasome 26 S subunit ATPase1^56^, PEX19^30–32^ and ribosomal protein L10a^57^, respectively. Of these, PEX19 is recently shown to mediate the posttranslational targeting of UBXD8, the membrane protein destined for lipid droplets^29^. Therefore, the identification of PEX19 prompted us to examine whether the RHD-containing proteins employed PEX19 for the posttranslational targeting in a similar manner to the lipid droplet-destined membrane.

We examined whether PEX19 selectively recognized the RHD-containing proteins. FLAG-Arl6IP1, FLAG-tagged Rtn4C (FLAG-Rtn4C) and FLAG-tagged reticulon 3 A (FLAG-Rtn3A) were synthesized *in vitro* with rabbit reticulocyte lysates as representatives of the RHD-containing proteins. Moreover, FLAG-tagged atlastin-1 (FLAG-atlastin-1) and FLAG-tagged syntaxin-1 (FLAG-syntaxin-1) were synthesized *in vitro* as a representative of other classes of the ER membrane-shaping proteins^58^ and a representative of the tail-anchored proteins^59^, respectively. After the translation reactions were terminated by puromycin, the *in vitro* synthesized FLAG-tagged proteins were pulled down with the anti-FLAG mAb-immobilized agarose, followed by Western blotting with the anti-PEX19 pAb. PEX19 was pulled down with FLAG-Rtn4C and FLAG-Rtn3A as well as FLAG-Arl6IP1 (Fig. 2B and Fig. S2). On the other hand, PEX19 was not pulled down with FLAG-syntaxin-1 and FLAG-atlastin-1 (Fig. 2B). These results indicate that PEX19 selectively recognizes the nascent RHD-containing proteins.

### PEX19 selectively mediates targeting of the RHD-containing proteins to the ER membrane

**Figure 3:**
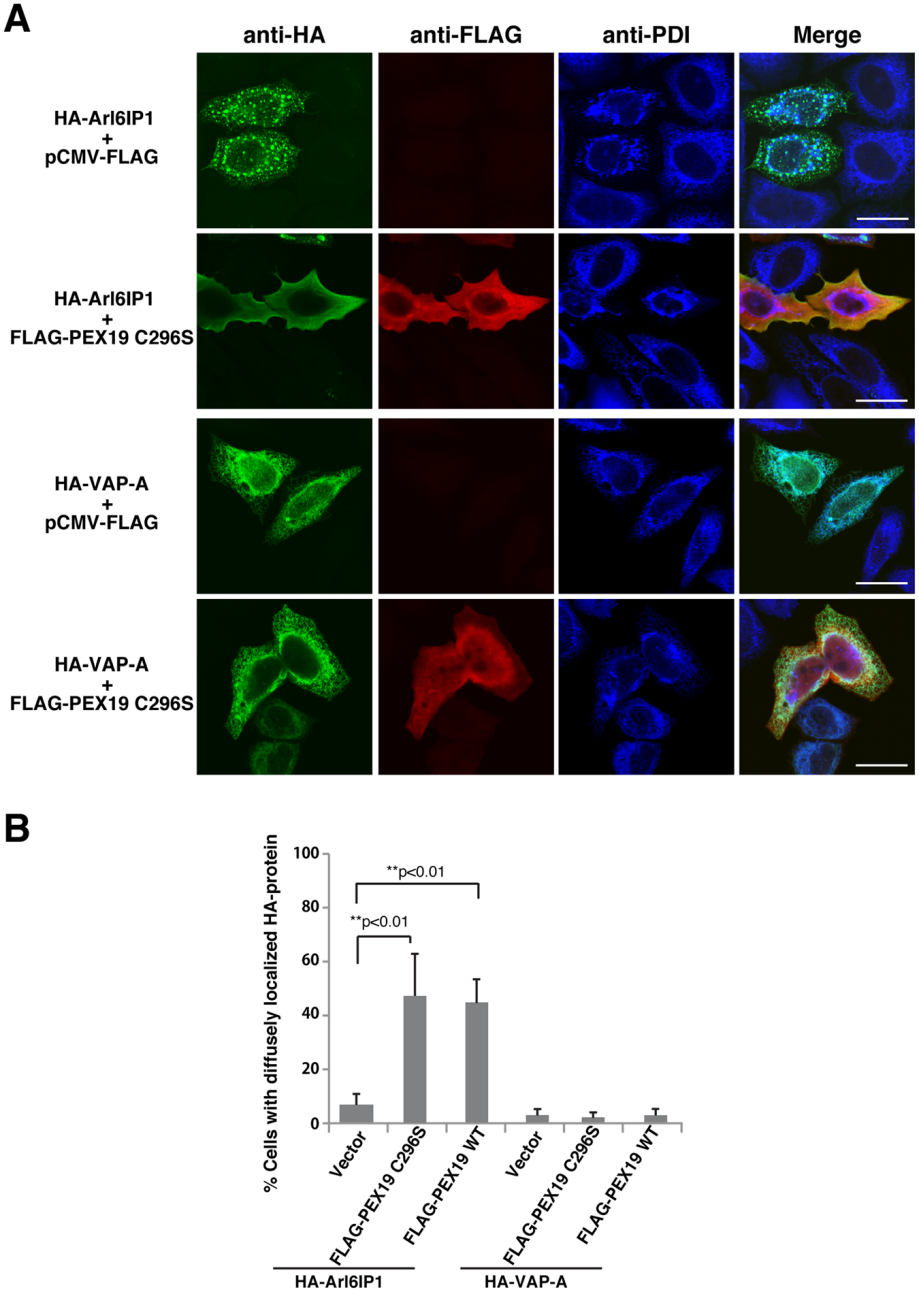
PEX19 selectively mediates targeting of Arl6IP1 to the ER membrane. (**A**) PEX19 overexpression-induced diffuse localization of Arl6IP1 but not VAP-A. HA-Arl6IP1 or HA-VAP-A was transfected into HeLa cells with or without FLAG-PEX19 C296S, followed by immunostaining with the anti-HA mAb, the anti-FLAG pAb and the anti-PDI mAb. Bars, 20 µm. (**B**) Quantification of the PEX19 overexpression-induced diffuse localization. 50 transfected cells were randomly chosen in (**A**) and the number of the cells showing the diffuse localization of HA-Arl6IP1 or HA-VAP-A was counted. HeLa cells were also transfected with HA-Arl6IP1 or HA-VAP-A and FLAG-wild-type PEX19 (FLAG-PEX19 WT) and immunostained in the same manner as in (**A**), and the number of the cells showing the diffuse localization of HA-Arl6IP1 or HA-VAP-A was likewise counted. The error bars represent SD of three independent experiments. Double asterisks indicate statistical significance (Student’s *t* test; **p < 0.01).

We next examined the role of PEX19 on targeting of the RHD-containing proteins to the ER membrane. The recent study has shown that farnesylated form of PEX19 is involved in the posttranslational targeting of UBXD8^29^. Therefore, we first examined the effect of overexpression of PEX19 C296S, the farnesylation-deficient mutant^60,61^ on targeting of Arl6IP1 to the ER membrane. HA-tagged Arl6IP1 (HA-Arl6IP1) was transfected into HeLa cells with or without FLAG-tagged PEX19 C296S (FLAG-PEX19 C296S). Consistent with our previous report^45^, overexpression of HA-Arl6IP1 showed the extensive reticular staining pattern indicative of the enhanced tubular ER network, induced the aberrant, circular structures of the ER, and excluded PDI from the peripheral ER tubules (Fig. 3A), indicating that membrane insertion of Arl6IP1 potently constricted the peripheral ER tubules. Importantly, co-overexpression of HA-Arl6IP1 and FLAG-PEX19 C296S frequently showed the diffuse staining of HA-Arl6IP1 (Fig. 3A,B), indicating that overexpression of PEX19 C296S induced aberrant diffuse localization of Arl6IP1. PDI was still excluded from the peripheral ER tubules in the cells overexpressing FLAG-PEX19 C296S, suggesting that a sufficient amount of Arl6IP1 was inserted into the ER membrane despite overexpression of PEX19 C296S. We next examined the effect of overexpression of wild-type PEX19. Of note, overexpression of FLAG-tagged wild-type PEX19 (FLAG-PEX19) exhibited the same effects as that of FLAG-PEX19 C296S (Fig. 3B). Therefore, these results indicate that overexpression of PEX19 interferes with targeting of a subpopulation of Arl6IP1 to the ER membrane. The similar results were obtained with Rtn3A. Overexpression of HA-tagged Rtn3A (HA-Rtn3A) showed the extensive tubular ER network and potently constricted the peripheral ER tubules as characterized by exclusion of PDI from the peripheral ER tubules (Fig. S3). Co-overexpression of HA-Rtn3A and FLAG-PEX19 C296S or FLAG-PEX19 frequently showed the diffuse staining of HA-Rtn3A, while PDI was still excluded from the peripheral ER tubules in the cells overexpressing FLAG-PEX19 C296S or FLAG-PEX19 (Fig. S3), indicating that overexpression of PEX19 interfered with targeting of a subpopulation of Rtn3A to the ER membrane. On the other hand, when HA-tagged VAP-A (HA-VAP-A), an ER-localized TA protein^62^ that is posttranslationally inserted by the TRC40 pathway, was transfected into HeLa cells with or without FLAG-PEX19 C296S or FLAG-PEX19, HA-VAP-A localized at the ER membrane regardless of overexpression of PEX19 and PEX19 C296S (Fig. 3A,B). Overexpression of PEX19 and PEX19 C296S also did not affect the ER localization of TMEM33, an ER membrane protein having three TMDs which was expected to be cotranslationally inserted by translocon^63^ (Fig. S4). Collectively, these results indicate that overexpression of PEX19 specifically interferes with targeting of the RHD-containing proteins to the ER membrane.

**Figure 4:**
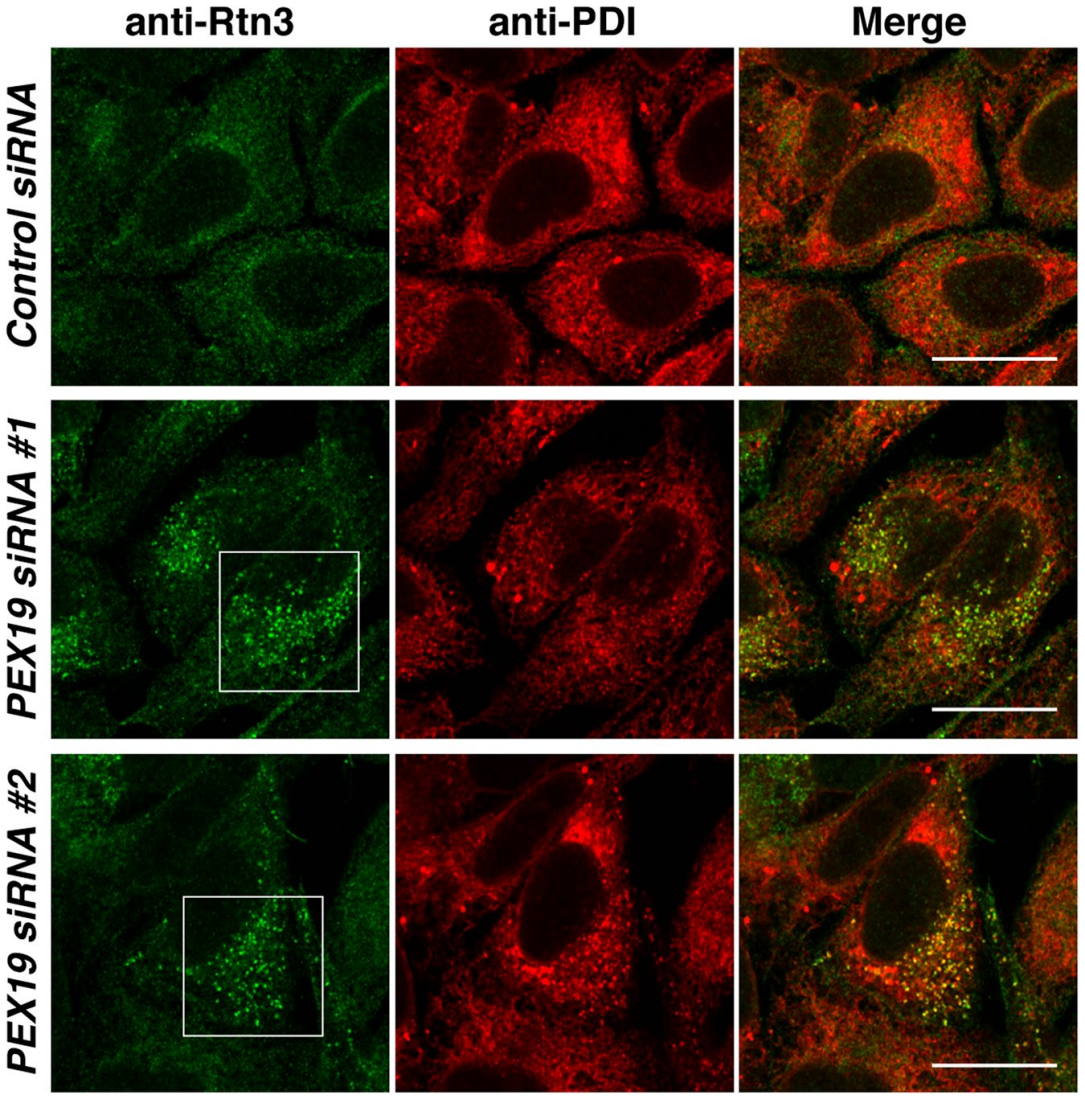
PEX19 knockdown causes cytoplasmic aggregation of endogenous reticulon 3 in the presence of MG132. HeLa cells were transfected with the siRNAs targeting PEX19 or the control siRNA and cultured in the presence of MG132, a proteasome inhibitor, followed by immunostaining with the anti-Rtn3 pAb and the anti-PDI mAb. Boxed areas indicate the dot-like immunoreactive signals suggestive of cytoplasmic aggregation of endogenous Rtn3. Bars, 20 µm.

We reasoned if PEX19 mediated targeting of the RHD-containing proteins to the ER membrane, depletion of PEX19 would cause accumulation of ER targeting-incompetent RHD-containing proteins aggregated in the cytoplasm. To test this possibility, we transfected the siRNAs targeting PEX19 or a negative control siRNA into HeLa cells. The knockdown of PEX19 was confirmed by immunoblotting (Fig. S5A). When the cells were cultured in the presence of MG132, a proteasome inhibitor, the dot-like immunoreactive signals for endogenous Rtn3 were frequently detected in the PEX19 knocked-down cells but not in the control siRNA-transfected cells (Fig. 4), suggesting that, in agreement with the above reasoning, aggregation of the membrane insertion-incompetent Rtn3 was caused in the cytoplasm by PEX19 knockdown. The dot-like immunoreactive signals were not detected in the absence of MG132 (Fig. S6), suggesting that the ER targeting-incompetent RHD-containing proteins were promptly degraded by the ubiquitin-proteasome system to avoid aggregation of the proteins in the cytoplasm.

We examined the effects of knockdown of the TRC40 pathway on the ER localization of Arl6IP1. The three independent siRNAs targeting CAML or WRB, both of which are the components of the TRC40 pathway^22–24^, were transfected into HeLa cells, followed by transfection of HA-Arl6IP1. Regardless of the knockdown of CAML or WRB, HA-Arl6IP1 localized at the ER membrane and deformed the ER membrane, indicating that the TRC40 pathway was not involved in PEX19-mediated ER targeting (Fig. S7**)**.

Overall, these results indicate that PEX19 selectively mediates targeting of the RHD-containing proteins to the ER membrane.

### PEX3 is involved in the posttranslational targeting of the RHD-containing proteins to the ER membrane

**Figure 5:**
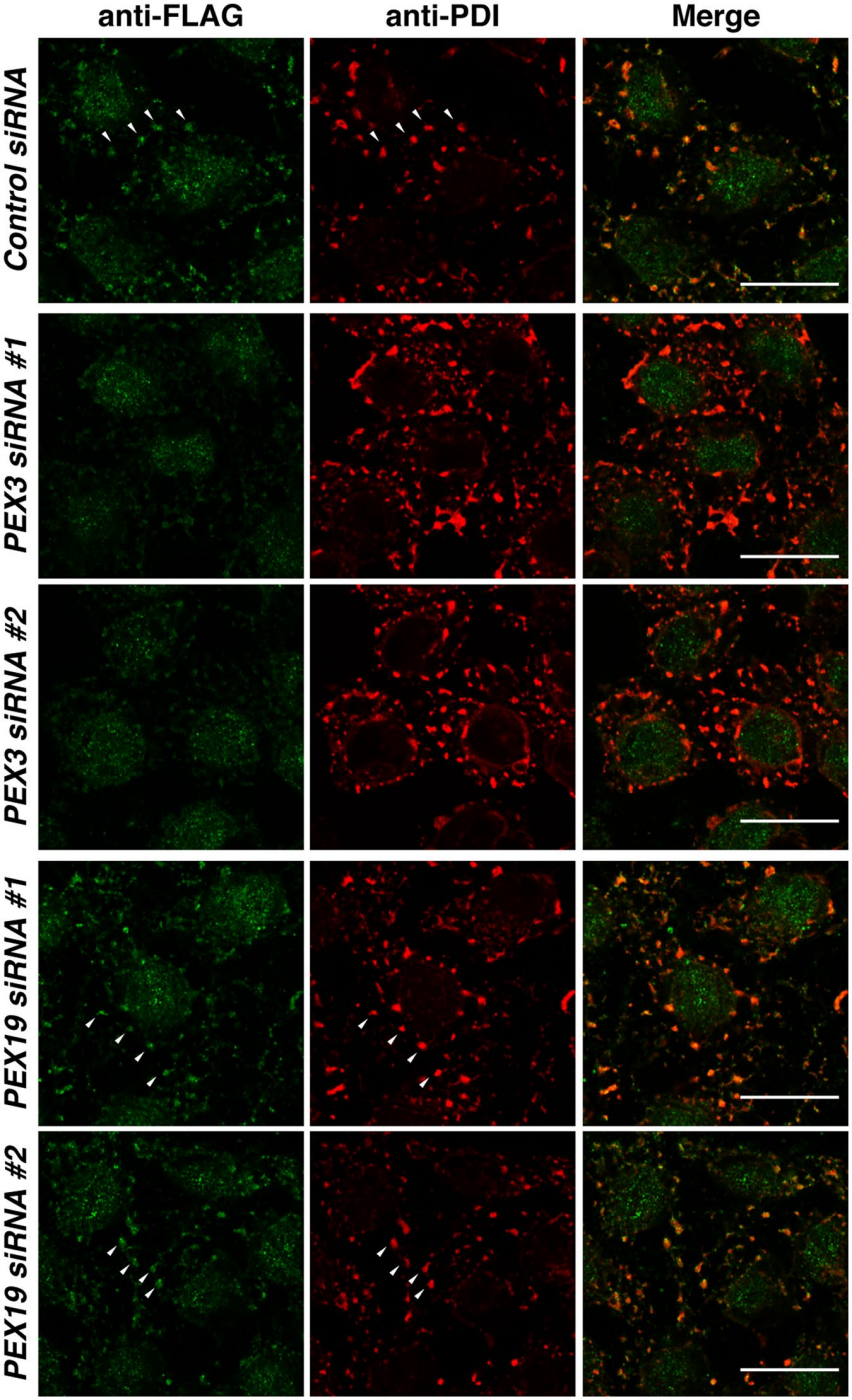
PEX3 is involved in the posttranslational targeting of Arl6IP1 to the ER membrane. HeLa cells were transfected with the siRNAs targeting PEX3 or PEX19 or the control siRNA, semi-permeabilized, and subjected to the *in vitro* posttranslational targeting assay with 3xFLAG-Arl6IP1 as in Fig. 1. Arrowheads indicate the posttranslational targeting to the ER membrane as judged by colocalization of 3xFLAG-Arl6IP1 with PDI. Bars, 20 µm.

PEX3 is the ER membrane receptor for PEX19^30–32^ and has been shown to mediate the posttranslational insertion of UBXD8 into the ER membrane in cooperation with PEX19^29^. We therefore examined whether PEX3 mediated the posttranslational targeting of the RHD-containing proteins into the ER membrane in a manner similar to the lipid droplet-destined membrane protein. The siRNAs targeting PEX3 and those targeting PEX19 were transfected into HeLa cells to allow knockdown of endogenous PEX3 and PEX19, respectively. The knockdown of PEX3 was confirmed by quantitative RT-PCR (Fig. S5B). The knocked-down cells were semi-permeabilized with digitonin and subjected to the *in vitro* posttranslational targeting assay with the rabbit reticulocyte lysates synthesizing 3xFLAG-Arl6IP1 or 3xFLAG-Rtn4C in the same manner as in Fig. 1. Consistent with the results in Fig. 1, the control siRNA-transfected cells showed strong immunoreactive signals for 3xFLAG-Arl6IP1 and 3xFLAG-Rtn4C at the ER, indicating that Arl6IP1 and Rtn4C were posttranslationally targeted to the ER membrane (Fig. 5 and Fig. S8). Importantly, the PEX3 knocked-down cells showed very weak immunoreactive signals for 3xFLAG-Arl6IP1 and 3xFLAG-Rtn4C at the ER relative to the control siRNA-transfected cells, indicating that the knockdown of PEX3 significantly decreased the posttranslational targeting. On the other hand, the immunoreactive signals for 3xFLAG-Arl6IP1 and 3xFLAG-Rtn4C in the PEX19 knocked-down cells were comparable to those in the control siRNA-transfected cells, presumably because the rabbit reticulocyte lysates supplied PEX19 to Arl6IP1 and Rtn4C in the *in vitro* posttranslational targeting assay. To validate the inhibitory effect of PEX3 knockdown on posttranslational targeting of the RHD-containing proteins, HA-tagged PEX3 constructs that had the silent mutations resistant to the siRNAs were transfected into the PEX3 knocked-down cells, followed by the *in vitro* posttranslational targeting assay. The PEX3 knocked-down cells transfected with siRNA-resistant PEX3-HA, which were identified by immunoreactivity for HA, showed stronger immunoreactive signals for 3xFLAG-Arl6IP1 and 3xFLAG-Rtn4C at the ER than the neighboring untransfected PEX3 knocked-down cells (Fig. S9), indicating that exogenous expression of PEX3 rescued the inhibitory effect of PEX3 knockdown. To further validate that PEX3 is specifically involved in posttranslational targeting of the RHD-containing proteins to the ER membrane, we examined the effect of knockdown of another peroxisome biogenesis factor. Two independent siRNAs targeting PEX5, which is essential for peroxisome biogenesis and mediates transport of peroxisomal matrix proteins to peroxisomes^31,64^, were transfected into HeLa cells to allow knockdown of endogenous PEX5 as confirmed by quantitative RT-PCR (Fig. S5C), followed by the *in vitro* posttranslational targeting assay. The PEX5 knocked-down cells showed significant immunoreactive signals for 3xFLAG-Arl6IP1 and 3xFLAG-Rtn4C, which were comparable to those in the cells transfeced with the control siRNA (Fig. S10), indicating that the ER targeting of the RHD-containing proteins was not affected by PEX5 knockdown. Overall, these results indicate that PEX3 specifically mediated posttranslational targeting of the RHD-containing proteins to the ER membrane, and concomitantly suggest that peroxisome biogenesis was not directly involved in the ER targeting of the RHD-containing proteins.

## Discussion

In this study, we show that RHD-containing proteins can take a posttranslational route for targeting to the ER membrane. PEX19 selectively recognizes the nascent RHD-containing proteins and mediates their posttranslational targeting in cooperation with PEX3. It has been established that the interaction between PEX19 and PEX3 constitutes the membrane insertion machinery for the peroxisomal membrane proteins and the lipid droplet-destined membrane proteins^29–32^. Therefore, it is plausible that the RHD-containing proteins guided by PEX19 will be inserted into the ER membrane by PEX3 interaction. However, our results do not deny the cotranslational route of the RHD-containing proteins, because we identified SRP54 as a protein that bound to the nascent RHD-containing proteins as shown in Fig. 2. Indeed, we observed that overexpression and knockdown of PEX19 were not sufficient to completely abolish the ER localization of the RHD-containing proteins as shown in Figs 3 and 4, raising the possibility that a significant number of the RHD-containing proteins might be cotranslationally inserted into the ER membrane. Besides, the previous study demonstrated that Rtn3 was cotranslationally targeted to the ER membrane^50^. Therefore, PEX19 and PEX3 will serve as an alternative membrane insertion machinery for the posttranslational route.

It remains unclear why the RHD-containing proteins need the two distinct routes for the membrane insertion. Given that RHD is usually located near the C-termini of the proteins, SRP seems likely to sometimes fail to recognize the RHD during translation. Therefore, one possible explanation is PEX19 and PEX3 may constitutively provide a backup route for the RHD-containing proteins that happen to miss the cotranslational route. Since PEX19 and PEX3 have been characterized as essential factors for peroxisome biogenesis^30–32^, the posttranslational route will be expected to be associated with peroxisome biogenesis. Of note, most of the RHD-containing proteins are the ER membrane-shaping proteins, raising that possibility that there may be functional coordination between ER membrane shaping and peroxisome biogenesis. In agreement, reticulons have been shown to be involved in regulation of the peroxisome biogenesis in yeasts^65,66^, although it remains unclear whether this is also the case with mammalian cells. Collectively, another possible explanation is that the RHD−containing proteins may preferentially take the posttranslational route to regulate the persoxisome biogenesis through shaping the ER membrane. In addition, given that PEX19 and PEX3 mediate the posttranslational membrane insertion of the lipid droplet-destined membrane proteins^29^, ER membrane shaping may be also associated with lipid droplet formation. Further studies will be required to understand how these three organelles, ER, peroxisomes and lipid droplets, communicate through the posttralnslational membrane insertion.

In this study, we demonstrated that the RHD-containing proteins were specific clients of PEX19 as shown in Fig. 2. It is widely accepted that RHD inserts the two short hairpin TMDs into the outer leaflet of membrane, inducing membrane curvature^38–40,44^. PEX19 has also been shown to mediate the posttranslational insertion of UBXD8 into the ER membrane^29^. Interestingly, UBXD8 has a single short hairpin TMD that is inserted into the outer leaflet of membrane, although it has no activity to induce membrane curvature. Taken together, these results raise an attractive possibility that PEX19 may preferentially recognize nascent short hairpin TMDs and mediate the posttranslational membrane insertion for not only the RHD-containing proteins and UBXD8 but also a broad range of membrane proteins harboring the short hairpin TMDs in cooperation with PEX3. Therefore, the PEX19-PEX3 system may serve as a fundamental machinery for insertion of any membrane proteins harboring the short hairpin TMDs into the ER membrane.

While it has been well established that PEX19 binds and inserts peroxisomal membrane proteins into the peroxisome membrane in cooperation with PEX3^30–32^, it remains unknown whether the short hairpin TMDs share the binding region of PEX19 with the peroxisomal membrane proteins. It also remains unclear whether farnesylation of PEX19 regulates binding to RHD. Further structural and biochemical analyses will be required to reveal the molecular mechanism how PEX19 recognizes a broad range of the short hairpin TMDs and mediates their membrane insertion.

In Fig. 3 and  Fig. S3, overexpression of wild-type PEX19 as well as that of PEX19 C296S induced aberrant diffuse localization of HA-Arl6IP1 and HA-Rtn3A. However, these results do not necessarily indicate that farnesylation of PEX19 is dispensable for ER targeting, because we have never examined how many overepressed wild-type PEX19 proteins are farnesylated. Therefore, we can not exclude the possibility that a significant fraction of overexpressed wild-type PEX19 may not be farnesylated, leading to induction of the aberrant diffuse localization of the RHD-containing proteins. On the other hand, there can be another possible explanation. Overexpressed PEX19 might catch cytoplasmic pools of HA-Arl6IP1 and HA-Rtn3A which should have been destined for proteasomal degradation due to a limited amount of endogenous PEX19. Indeed, cytoplasmic accumulation of endogenous Rtn3 was detected in the PEX19 knocked-down cells only in the presence of MG132, the proteasome inhibitor, as shown in Fig. 4, suggesting that the cytoplasmic pools of RHD-containing proteins are degraded and promptly removed by proteasome when the cells lack a sufficient amount of PEX19. Most of the PEX19–HA-Arl6IP1 and PEX19–HA-Rtn3A complexes might not be targeted to the ER membrane due to a limited amount of endogenous PEX3, eventually allowing them to aberrantly stay in the cytosol. Further studies will be required to reveal involvement of farnesylation of PEX19 in ER targeting of the RHD-containing proteins and to clarify the effects of overexpression of PEX19.

In addition to PEX19, we purified Bat3 from the rabbit reticulocyte lysates as a protein that bound to nascent Arl6IP1 as shown in Fig. 2. Interestingly, the previous study has also demonstrated that Bat3 is purified from the rabbit reticulocyte lysates as a protein that bound to nascent UBXD8^29^. Therefore, there is the possibility that Bat3 may regulate the PEX19-mediated recognition of short hairpin TMDs. On the other hand, Bat3 has been shown to capture the TMDs of mislocalized membrane proteins to prevent undesired aggregation in the cytosol, leading to their degradation through ubiquitin-proteasome system^54,55^. Therefore, the possibility can not be also ruled out that Bat3 may bind to the short hairpin TMDs of ER targeting-incompetent RHD-containing proteins or UBXD8 toward the proteasomal degradation. In agreement, the proteasome inhibitor was required to detect the cytoplasmic aggregation of Rtn3 as shown in Fig. 4, suggesting ER targeting-incompetent Rtn3 was the substrate for the ubiquitin-proteasome system. Further biochemical analyses will be required to address these concerns.

Overall, the peroxisome biogenesis factors, PEX19 and PEX3, provide an alternative posttranslational route for ER targeting of the RHD-containing proteins, implying that ER membrane shaping may coordinate with peroxisome biogenesis through membrane insertion, and that the peroxisome biogenesis factors may serve as a fundamental machinery for insertion of a broad range of membrane proteins harboring short hairpin TMDs into the ER membrane.

## Materials and Methods

### Plasmids

For *in vitro* translation, the cDNAs encoding human Arl6IP1, human reticulon 4C (Rtn4C), mouse reticulon 3A (Rtn3A), rat atlastin-1 and rat syntaxin-1 were subcloned into the pBluescript KS vectors under the control of the T7 promoter with the N-terminal triple FLAG tags (3xFLAG) or single FLAG tag. For mammalian expression, the cDNAs encoding human Arl6IP1, mouse Rtn3A, rat VAP-A, human PEX19 and the farnesylation-deficient mutant of human PEX19 (PEX19 C296S) were subcloned into the pCMV vectors with the N-terminal HA tag or FLAG tag. The cDNAs encoding human PEX3 and human TMEM33 were subcloned into the pCA vectors with the C-terminal HA tag. To generate the siRNA-resistant forms of PEX3, the silent mutations were introduced by site-directed mutagenesis using QuikChange Lightning (Agilent Technologies) with the following primer sets. The sense primer for siRNA #1-resistant PEX3; 5′-ctcaatgaaactagagacatgttggaaagccctgacttctccacagttttgaatacctgtttaaaccgaggttt-3′. The antisense primer for siRNA #1-resistant PEX3; 5′-aaacctcggtttaaacaggtattcaaaactgtggagaagtcagggctttccaacatgtctctagtttcattgag-3′. The sense primer for siRNA #2-resistant PEX3; 5′-ggaggctgcagaatacattgcccaagctaggaggcagtatcattttgaaagtaaccagaggactt-3′. The antisense primer for siRNA #2-resistant PEX3; 5′-aagtcctctggttactttcaaaatgatactgcctcctagcttgggcaatgtattctgcagcctccc-3′.

### Antibodies

A rabbit anti-PEX19 pAb, a mouse anti-PDI mAb, a rabbit anti-Rtn3 pAb, a rat anti-HA mAb and a rabbit anti-FLAG pAb were purchased from Thermo Fisher Scientific (Cat. No. PA5–22129), Abcam (Cat. No. ab2792), Proteintech (Cat. No. 12055–2-AP), Roche (Cat. No. 11 867 423 001) and Sigma (Cat. No. F7425), respectively.

### Preparation of semi-intact HeLa cells

HeLa cells were grown on 12 mm coverslips and semi-permeabilized with digitonin as described previously^23^. Briefly, HeLa cells grown on coverslips were rinsed with ice-cold KHM buffer containing 20 mM HEPES–KOH pH7.4, 110 mM potassium acetate (KOAc), 2 mM magnesium acetate and permeabilized with KHM buffer supplemented with 40 µg/ml digitonin on ice for 10 min. The cells were washed with KHM buffer once, followed by HEPES buffer containing 90 mM HEPES–KOH pH7.4 and 50 mM KOAc twice. The cells were treated with micrococcal nuclease (New England BioLabs) in HEPES buffer supplemented with 2.5 mM CaCl_2_ at room temperature for 20 min. Following washing twice with KHM buffer, the semi-permeabilized cells on the coverslips were subjected to the *in vitro* membrane targeting assay as described below.

In some instances, 10 nM of the silencer-select siRNAs targeting human PEX19 (Thermo Fisher Scientific; Cat. No. s11612, s11613), human PEX3 (Thermo Fisher Scientific; Cat. No. s16154, s16156) or human PEX5 (Thermo Fisher Scientific; Cat. No. s11630, s11632) were transfected into HeLa cells with Lipofectamine RNAi MAX (Invitrogen) and cultured for 48–72 h to allow knockdown of PEX19, PEX3 or PEX5, followed by semi-permeabilization. Of note, these siRNAs are the same as those validated in the previous study^29^.

### *In vitro* assay for posttranslational targeting of membrane proteins to the ER membrane

3xFLAG-Arl6IP1 and 3xFLAG-Rtn4C were synthesized *in vitro* with the rabbit reticulocyte lysate-based transcription–translation coupled system (TnT Quick Coupled Transcription/Translation System (Promega)) using pBluescript KS-3xFLAG-Arl6IP1 and pBluescript KS-3xFLAG-Rtn4C as templates, respectively, in accordance with the manufacturer’s manual. The translation reactions were terminated by addition of 1 mM cycloheximide. The semi-permeabilized HeLa cells on the coverslips were incubated with 35 µl of the rabbit reticulocyte lysates synthesizing 3xFLAG-Arl6IP1 or 3xFLAG-Rtn4C at 37 °C for 90 min to allow the posttranslational targeting into the ER membrane. After being washed with KHM buffer three times, the cells were fixed with 4% paraformaldehyde, followed by permeabilization with 0.2% Triton X-100. In some instances, after being washed with KHM buffer three times, the cells were incubated with 0.1 M sodium carbonate (pH11.0) at 4 °C for 30 min to extract peripheral membrane proteins and washed again with KHM buffer three times, followed by fixation and permeabilization. The samples were doubly immunostained with the anti-FLAG pAb and the anti-PDI mAb as described below in Immunohistochemistry.

### Biochemical assessment of membrane insertion of the RHD-containing proteins by sodium carbonate extraction

HeLa cells were grown on 6-cm dishes and semi-permeabilized with digitonin in the same manner as described above and suspended in KHM buffer. 3xFLAG-Arl6IP1 and 3xFLAG-Rtn4C were synthesized *in vitro* with 200 µl of the rabbit reticulocyte lysates as described above. After the translation reactions were terminated by addition of 1 mM cycloheximide, the rabbit reticulocyte lysates were mixed with 25 µl (110 µg of the proteins) of the semi-permeablilized cell suspension in microcentrifuge tubes and rotate at 37 °C for 90 min to allow the posttranslational targeting into the ER membrane. To remove the rabbit reticulocyte lysates containing uninserted 3xFLAG-Arl6IP1 and 3xFLAG-Rtn4C, the semi-permeablilized cells were collected by centrifugation at 1,200 × *g* at 4 °C for 5 min. The semi-permeablilized cells were resuspended in KHM buffer and centrifuged at 1,200 × *g* at 4 °C for 5 min. To completely remove the rabbit reticulocyte lysates, this wash step was repeated three times. Then the semi-permeablilized cells were suspended in 100 µl of 0.1 M sodium carbonate (pH11.0) and incubated at 4 °C for 30 min to extract peripheral membrane proteins, followed by untracentrifugton at 100,000 × *g* at 4 °C for 30 min. The supernatant (extracted fraction) was subjected to TCA precipitation and resuspended in 120 µl of SDS sample buffer. The pellet (unextracted fraction) was resuspended in 120 µl of SDS sample buffer.

### Immunoaffinity purification of Arl6IP1-binding proteins

FLAG-Arl6IP1 was synthesized *in vitro* with TnT Quick Coupled Transcription/Translation System using pBluescript KS-FLAG-Arl6IP1 as a template. 250 µl of the rabbit reticulocyte lysate synthesizing FLAG-Arl6IP1 was incubated with 30 µl of anti-FLAG M2 mAb-conjugated agarose beads (Sigma) at 4 °C for overnight. After being washed extensively with binding buffer containing 20 mM Tris–HCl pH7.5, 150 mM NaCl, 2 mM MgCl_2_, the bound proteins were eluted with binding buffer supplemented with 0.3 mg/ml FLAG peptide. The samples were subjected to SDS-PAGE followed by silver staining. The bands of interest were cut out from the gel and digested with trypsin, followed by mass spectrometry analysis.

### Binding of PEX19 to the RHD-containing proteins

FLAG-Arl6IP1, FLAG-Rtn3A, FLAG-Rtn4C, FLAG-atlastin-1 and FLAG-syntaxin-1 were synthesized *in vitro* with TnT Quick Coupled Transcription/Translation System using pBluescript KS-FLAG-Arl6IP1, pBluescript KS-FLAG-Rtn3A, pBluescript KS-FLAG-Rtn4C, pBluescript KS-FLAG-atlastin-1 and pBluescript KS-FLAG-syntaxin-1 as templates, respectively, followed by addition of 1 mM puromycin to terminate the translation reaction. 80 µl of the rabbit reticulocyte lysate was incubated with 10 µl of anti-FLAG M2 mAb-conjugated agarose beads at 4 °C for 4 h. After being washed extensively with binding buffer, the bound proteins were eluted with an SDS sample buffer with boiling. The samples were subjected to SDS-PAGE followed by Western blotting with the anti-PEX19 pAb and the anti-FLAG pAb.

### Immunohistochemistry

Appropriate combinations of the mammalian expression vectors were transfected into the cells cultured on 12 mm coverslips with Effectene (QIAGEN). The day after transfection, the cells were fixed with 4% paraformaldehyde, followed by permeabilization with 0.2% Triton X-100. After blocking with PBS containing 1% BSA, the samples were incubated with primary Abs, followed by incubation with secondary antibodies conjugated with Alexa Fluor dyes (Invitrogen) for 30 min. After being washed with PBS, they were embedded and viewed using a confocal imaging system (ZEISS, LSM 510 Meta).

For knockdown of PEX19, 10 nM of the silencer-select siRNAs targeting human PEX19 were transfected into HeLa cells with Lipofectamine RNAi MAX. The day after transfection, medium was replaced with fresh one supplemented with 1 µM MG132 (Sigma), and the cells were cultured for 17 h to allow inhibition of proteasome, followed by immunostaining as described above.

For knockdown of CAML or WRB, 50 nM of the stealth siRNAs targeting human CAML (Thermo Fisher Scientific; Cat. No. CAMLGHSS188680, CAMLGHSS188681, CAMLGHSS101333) or WRB (Thermo Fisher Scientific; Cat. No. WRBHSS111382, WRBHSS111383, WRBHSS111384) were transfected into HeLa cells with Lipofectamine RNAi MAX. The day after transfection, HA-Arl6IP1 was subsequently transfected with Effectene and further cultured overnight to allow CAML or WRB to be fully knocked down and expression of HA-Arl6IP1, followed by immunostaining as described above. Of note, these siRNAs have been already validated in our previous study^23^.

### Quantitative RT-PCR

Total RNAs were isolated from HeLa cells transfected with siRNAs of interest using the Qiagen RNeasy Mini kit (QIAGEN) and reverse transcribed with SuperScript VILO (Invitrogen) into the cDNAs. Quantitative PCR was performed on LightCycler^®^ 480 Real Time PCR System (Roche) using KAPA SYBR FAST Master Mix (KAPA BIOSYSTEMS). The quantities of PEX3 transcripts and PEX5 transcripts were normalized to GAPDH.

## Electronic supplementary material

Supplementary figures
