# Supplementary material for: The peroxisome biogenesis factors posttranslationally target reticulon homology domain-containing proteins to the endoplasmic reticulum membrane
Source: Sci Rep. 2018 Feb 2;8:2322. doi: 10.1038/s41598-018-20797-0 (PMC5797116; doi:10.1038/s41598-018-20797-0)

Supplementary information

The peroxisome biogenesis factors posttranslationally target reticulon homology domain-containing proteins to the endoplasmic reticulum membrane.

Yasunori Yamamoto, Toshiaki Sakisaka

Division of Membrane Dynamics, Department of Physiology and Cell Biology, Kobe University Graduate School of Medicine, Kobe 650-0017, Japan

Address correspondence to Toshiaki Sakisaka, Division of Membrane Dynamics, Department of Physiology and Cell Biology, Kobe University Graduate School of Medicine, Kobe 650-0017, Japan
Tel.: +81-78-382-5727
Fax: +81-78-382-5419
E-mail: sakisaka@med.kobe-u.ac.jp

Supplementary figure legends

Figure S1. The RHD-containing proteins that are posttranslationally targeted to the ER membrane are resistant to sodium carbonate extraction.

(**A**) Immunocytochemical assessment of resistance to sodium carbonate extraction. The rabbit reticulocyte lysate synthesizing 3xFLAG-Arl6IP1 or 3xFLAG-Rtn4C or the rabbit reticulocyte lysate without the 3xFLAG-tagged proteins was supplemented with 1 mM cycloheximide and incubated with the semi-permeabilized HeLa cells on the coverslip to allow posttranslational targeting of the 3xFLAG-tagged proteins to the ER membranes. After being washed with buffer, the semi-permeabilized cells were incubated with 0.1 M sodium carbonate (pH11.0) at 4 °C for 30 min to extract the peripheral membrane proteins, followed by immunostaining with the anti-FLAG pAb and the anti-PDI mAb. Bars, 20 μm.

(**B**) Biochemical assessment of resistance to sodium carbonate extraction. The rabbit reticulocyte lysate synthesizing 3xFLAG-Arl6IP1 or 3xFLAG-Rtn4C was supplemented with 1 mM cycloheximide and incubated with the semi-permeabilized HeLa cell suspension in the microcentrifuge tube to allow posttranslational targeting of the 3xFLAG-tagged proteins to the ER membranes. After being washed with buffer, the semi-permeabilized HeLa cells were suspended in 0.1 M sodium carbonate (pH11.0) and incubated at 4 °C for 30 min to extract the peripheral membrane proteins, followed by ultracentrifugation at 100,000 ×*g* at 4 °C for 30 min. The supernatant and the pellet were subjected to Western blotting with the anti-FLAG pAb and the anti-PDI mAb.

Figure S2. Binding of PEX19 to nascent reticulon 3A.

The rabbit reticulocyte lysate synthesizing FLAG-Rtn3A was supplemented with 1 mM cycloheximide, followed by incubation with anti-FLAG mAb-immobilized agarose. The bound proteins were eluted with the SDS sample buffer with boiling and subjected to SDS-PAGE followed by Western blotting with the anti-PEX19 pAb and the anti-FLAG pAb.

Figure S3. PEX19 mediates targeting of reticulon 3A to the ER membrane.

(A) PEX19 overexpression-induced diffuse localization of Rtn3A. HA-Rtn3A was transfected into HeLa cells with or without FLAG-wild-type PEX19 (FLAG-PEX19

WT) or FLAG-PEX19 C296S, followed by immunostaining with the anti-HA mAb, the anti-FLAG pAb and the anti-PDI mAb. Arrowheads indicate the cells showing diffuse localization of HA-Rtn3A. Bars, 20 μm.

(B) Quantification of the PEX19 overexpression-induced diffuse localization of Rtn3A. 50 transfected cells were randomly chosen in **(A)** and the number of the cells showing the diffuse localization of HA-Rtn3A was counted. The error bars represent SD of three independent experiments. Double asterisks indicate statistical significance (Student's *t* test; **, p <0.01).

Figure S4. PEX19 is not involved in ER localization of TMEM33.

(A) No effect of overexpression of PEX19 on ER localization of TMEM33. TMEM33-HA was transfected into HeLa cells with or without FLAG-PEX19 C296S or FLAG-PEX19 WT, followed by immunostaining with the anti-HA mAb, the anti-FLAG pAb and the anti-PDI mAb. Bars, 20 μm.

(B) Quantification of the effect of overexpression of PEX19 on ER localization of TMEM33. 50 transfected cells were randomly chosen in **(A)** and the number of the cells showing the diffuse localization of TMEM33-HA was counted. The error bars represent SD of three independent experiments.

Figure S5. Validation of siRNA-mediated knockdowns of PEX19, PEX3 and PEX5.

(A) Validation of PEX19 knockdown by Western blotting. HeLa cells were transfected with the siRNAs targeting PEX3 or PEX19 or the control siRNA. The total cell lysates were subjected to SDS-PAGE followed by Western blotting with the anti-PEX19 pAb.

(B) Validation of PEX3 knockdown by quantitative RT-PCR. Total RNAs were isolated from HeLa cells transfected with the siRNAs targeting PEX3 or PEX19 or the control siRNA and reverse transcribed into the cDNAs, followed by quantitative RT-PCR. The quantities of PEX3 transcripts were shown relative to GAPDH. The error bars represent SD of three independent measurements.

(C) Validation of PEX5 knockdown by quantitative RT-PCR. Total RNAs were isolated from HeLa cells transfected with the siRNAs targeting PEX5 or the control siRNA and reverse transcribed into the cDNAs, followed by quantitative RT-PCR. The quantities of PEX5 transcripts were shown relative to GAPDH. The error bars represent SD of three independent measurements.

Figure S6. PEX19 knockdown has no effect on ER localization of reticulon 3 in the absence of MG132.
HeLa cells were transfected with the siRNAs targeting PEX19 or the control siRNA and cultured in the absence of MG132, a proteasome inhibitor, followed by immunostaining with the anti-Rtn3 pAb and the anti-PDI mAb. Bars, 20 μm.

Figure S7. The TRC40 pathway is not involved in ER localization of Arl6IP1.
HeLa cells were transfected with the siRNAs targeting CAML or WRB or the control siRNA and cultured overnight. HA-Arl6IP1 was subsequently transfected and further cultured overnight to allow CAML or WRB to be fully knocked down and expression of HA-Arl6IP1, followed by immunostaining with the anti-HA mAb and the anti-PDI mAb. Bars, 20 μm.

Figure S8. PEX3 is involved in the posttranslational targeting of reticulon 4C to the ER membrane.
HeLa cells were transfected with the siRNAs targeting PEX3 or PEX19 or the control siRNA, semi-permeabilized, and subjected to the *in vitro* posttranslational targeting assay with 3xFLAG-Rtn4C as in **Figure 1**. Arrowheads indicate the posttranslational targeting to the ER membrane as judged by colocalization of 3xFLAG-Rtn4C with PDI. Bars, 20 μm.

Figure S9. Rescue of the effect of knockdown of PEX3 on posttranslational targeting of the RHD-containing proteins by exogenous expression of PEX3.
(A) Rescue of the effect of knockdown of PEX3 on posttranslational targeting of Arl6IP1. HeLa cells were transfected with the siRNAs targeting PEX3 or the control siRNA and cultured overnight. The cells were subsequently transfected with siRNA-resistant PEX3-HA and further cultured overnight to allow PEX3 to be fully knocked down and expression of siRNA-resistant PEX3-HA. The cells were then semi-permeabilized and subjected to the *in vitro* posttranslational targeting assay with 3xFLAG-Arl6IP1 in the same manner as in **Figure 1** except that the cells were immunostained with the anti-FLAG pAb, the anti-PDI mAb and the anti-HA mAb.

Arrowheads indicate the ER targeting of 3xFLAG-Arl6IP1 which was restored by expression of siRNA-resistant PEX3-HA. Bars, 20 μm.

(B) Rescue of the effect of knockdown of PEX3 on posttranslational targeting of reticulon 4C. The experiments were carried out in the same manner as in **(A)** except that 3xFLAG-Rtn4C was used. Arrowheads indicate the ER targeting of 3xFLAG-Rtn4C which was restored by expression of siRNA-resistant PEX3-HA. Bars, 20 μm.

Figure S10. PEX5 is not involved in the posttranslational targeting of the RHD-containing proteins to the ER membrane.

(A) No effect of PEX5 knockdown on posttranslational targeting of Arl6IP1. HeLa cells were transfected with the siRNAs targeting PEX5 or the control siRNA, semi-permeabilized, and subjected to the *in vitro* posttranslational targeting assay with 3xFLAG-Arl6IP1 as in **Figure 1**. Arrowheads indicate the posttranslational targeting to the ER membrane as judged by colocalization of 3xFLAG-Arl6IP1 with PDI. Bars, 20 μm.

(B) No effect of PEX5 knockdown on posttranslational targeting of reticulon 4C. The experiments were carried out in the same manner as in **(A)** except that 3xFLAG-Rtn4C was used. Arrowheads indicate the posttranslational targeting to the ER membrane as judged by colocalization of 3xFLAG-Rtn4C with PDI. Bars, 20 μm.

Figure S11. Uncropped western blots for Figure 2B, Figure S1B, Figure S2 and Figure S5A.

Boxes indicate cropped areas.

Supplementary Figure 1

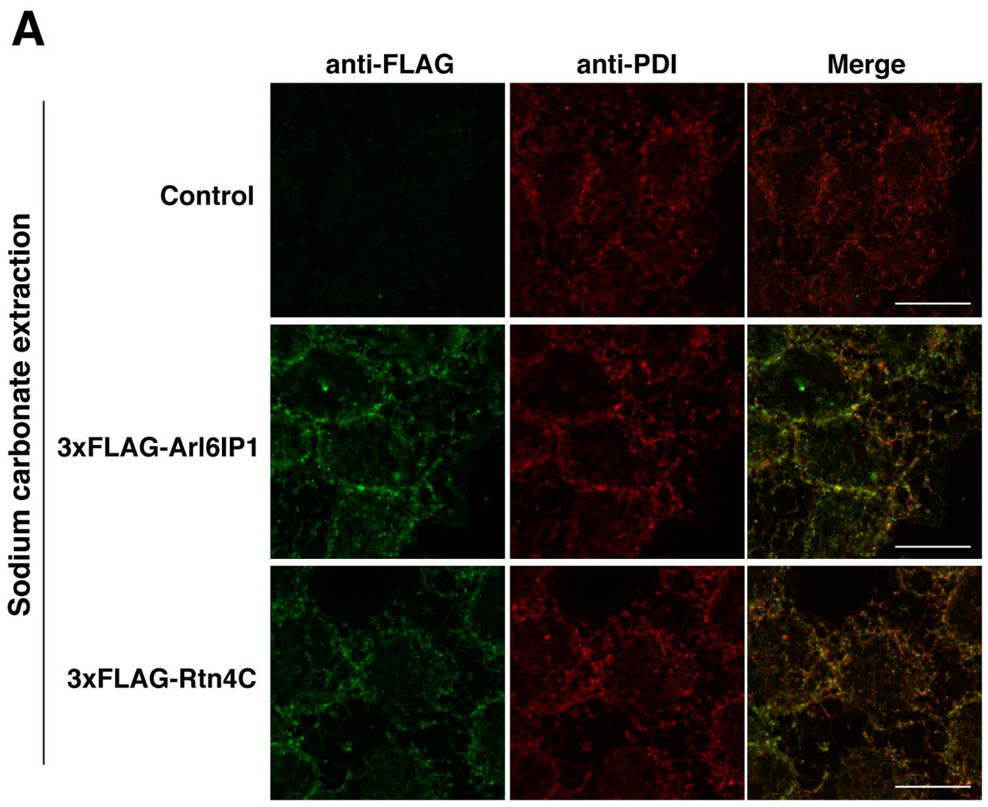

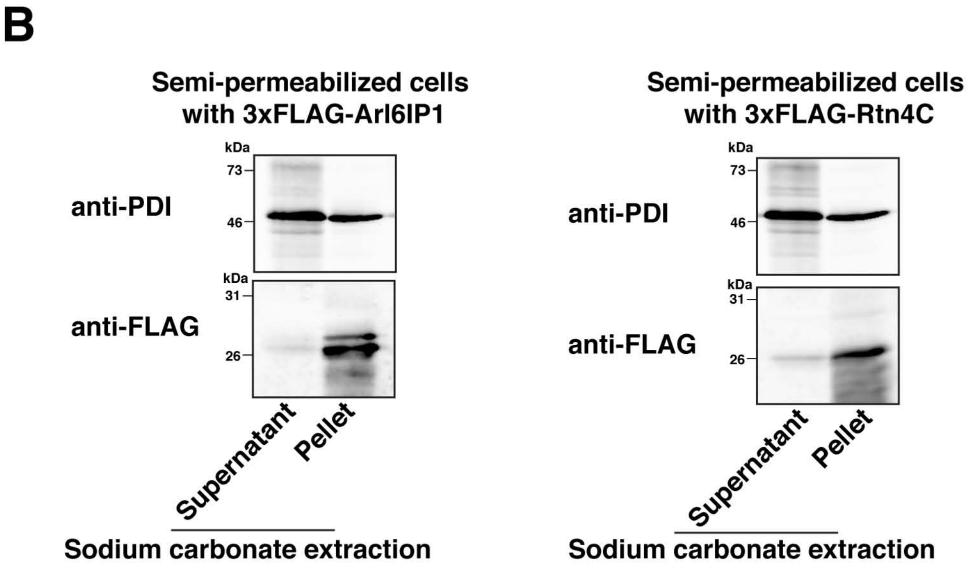

Supplementary Figure 2

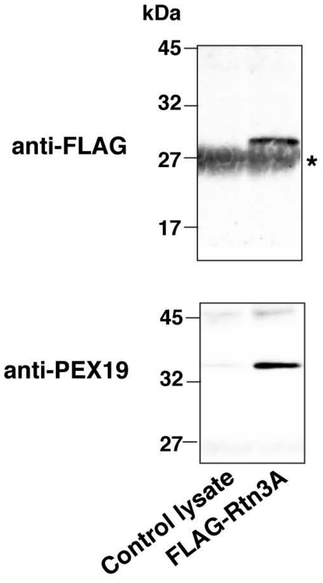

Supplementary Figure 3

A

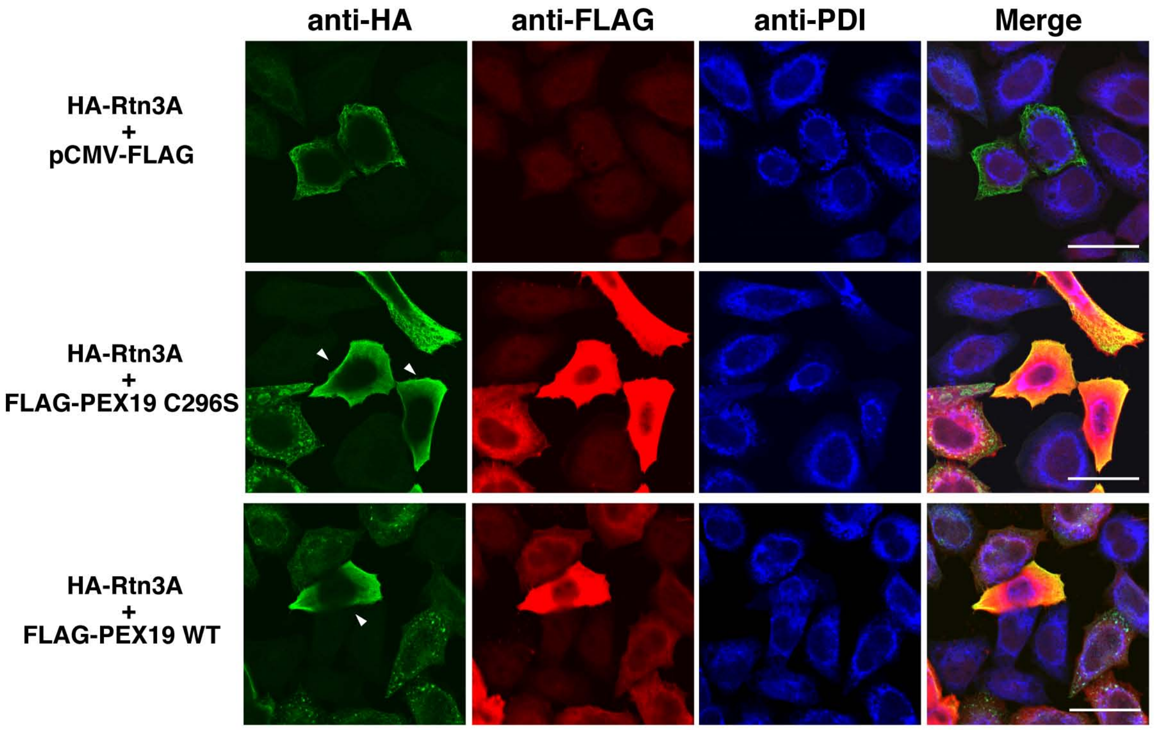

B

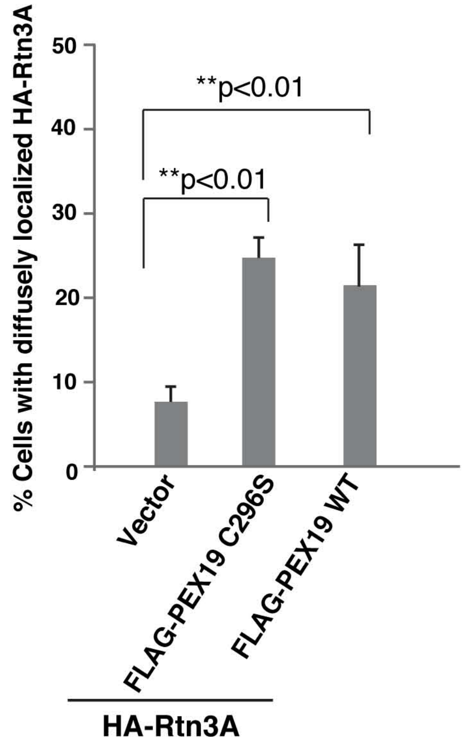

Supplementary Figure 4

A

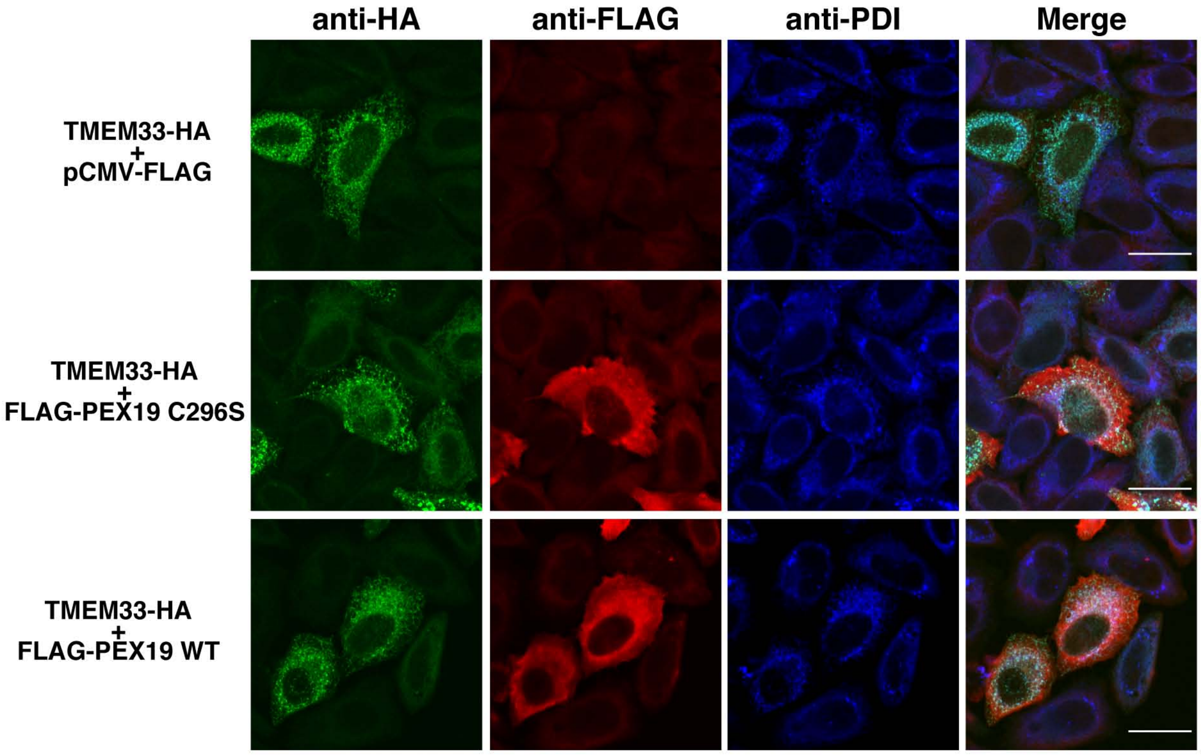

B

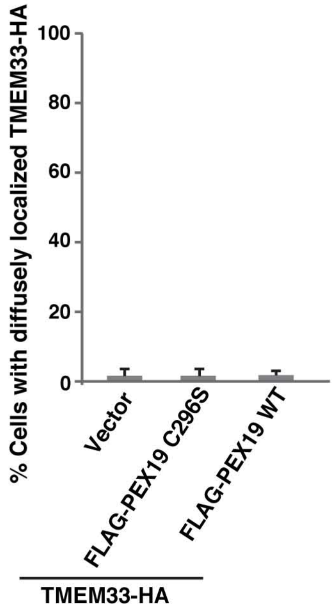

Supplementary Figure 5

A

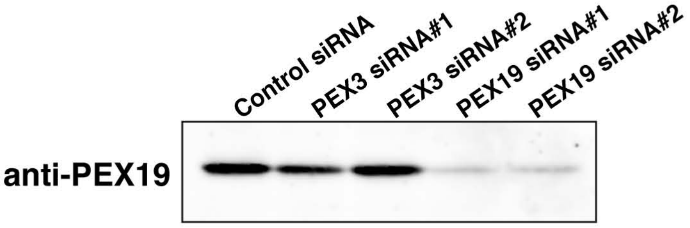

B

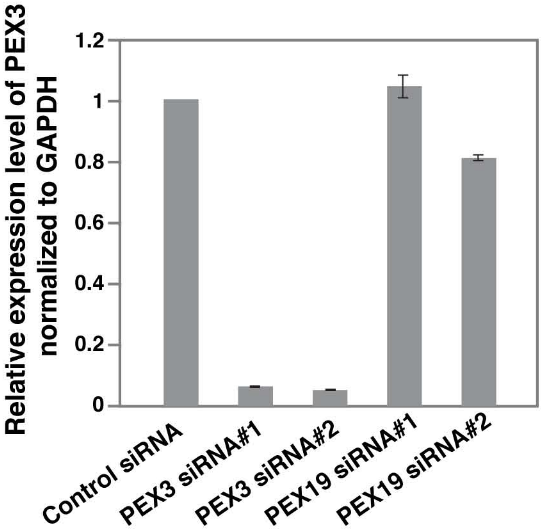

C

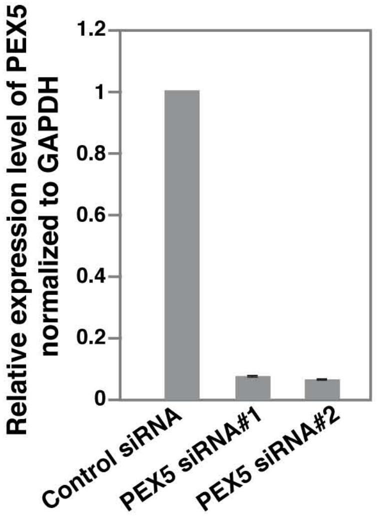

Supplementary Figure 6

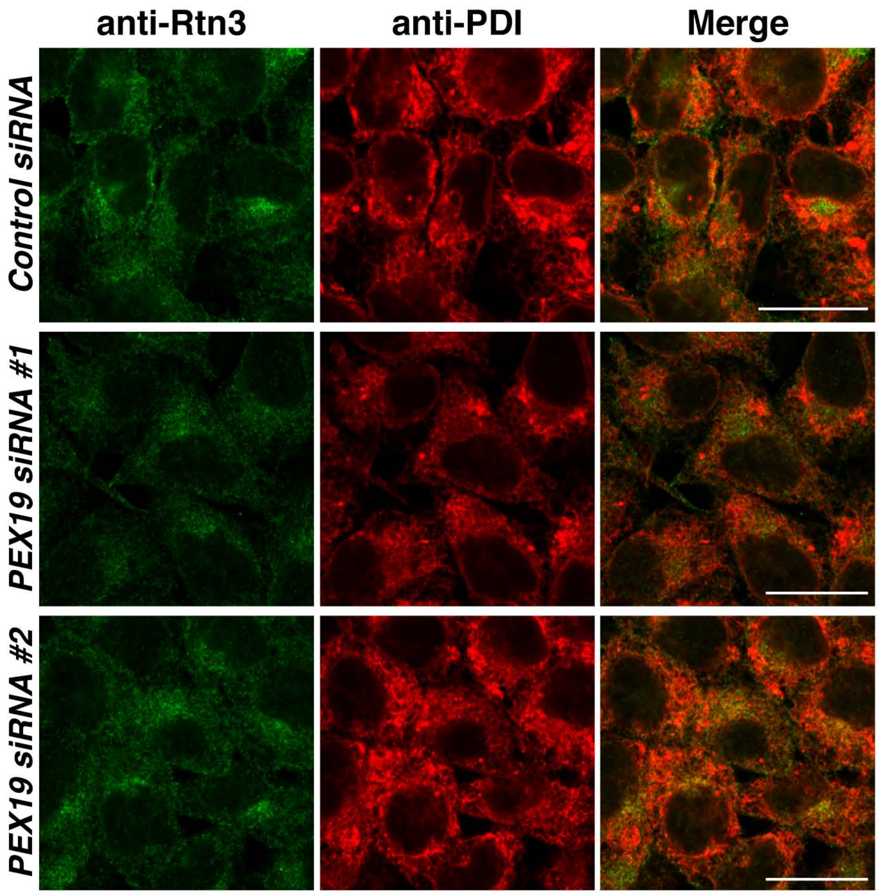

Supplementary Figure 7

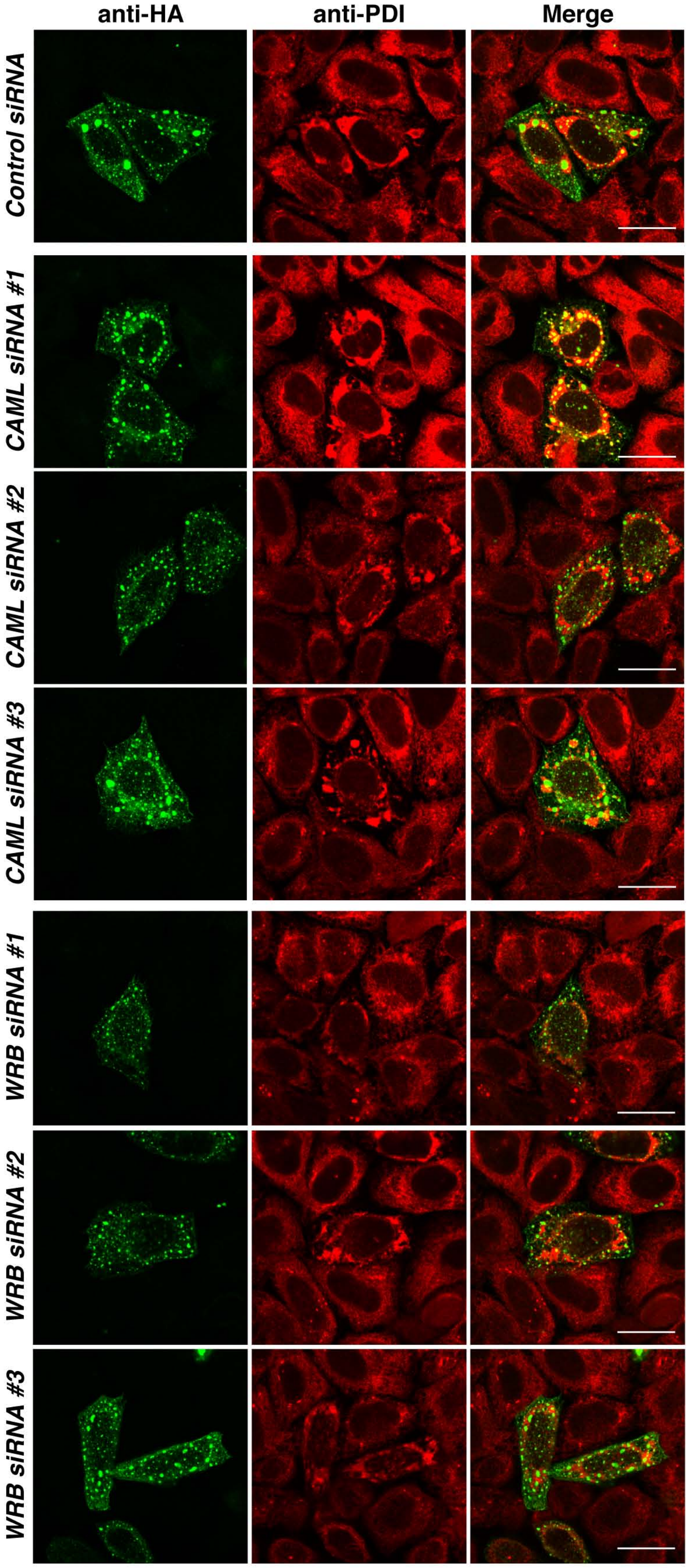

Supplementary Figure 8

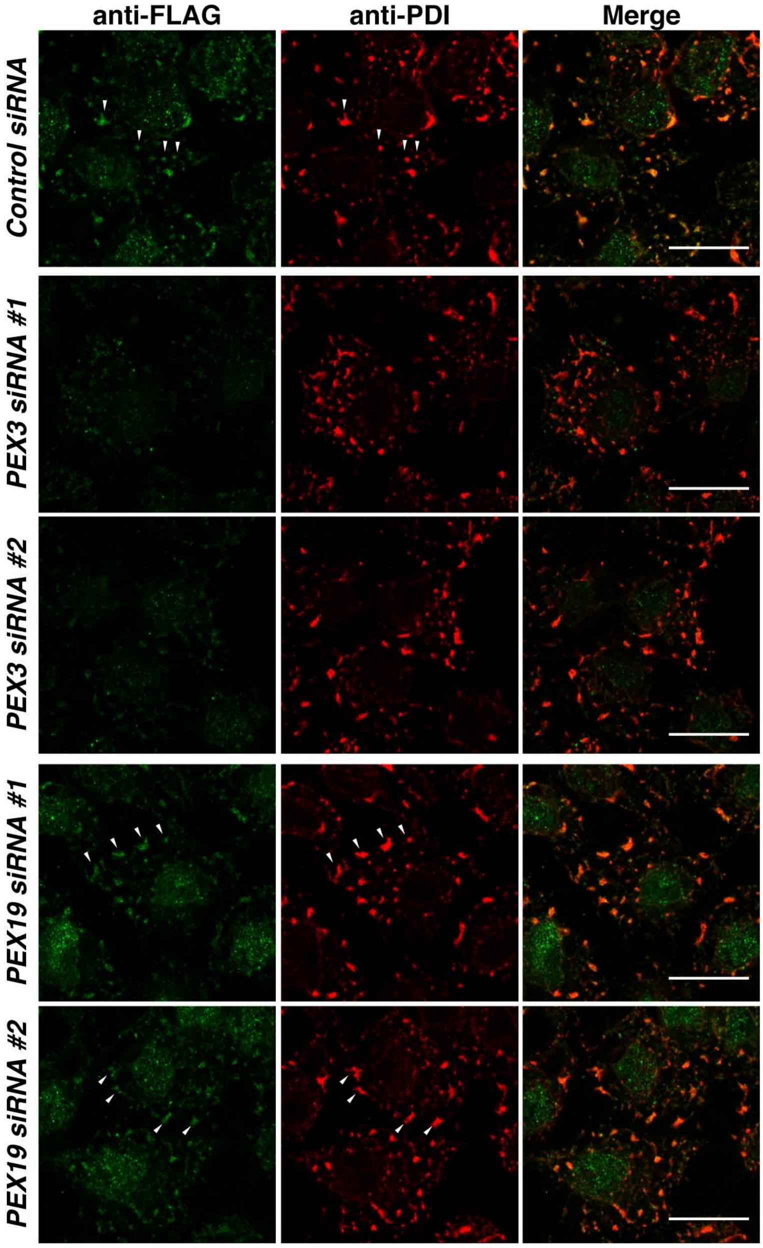

Supplementary Figure 9

A

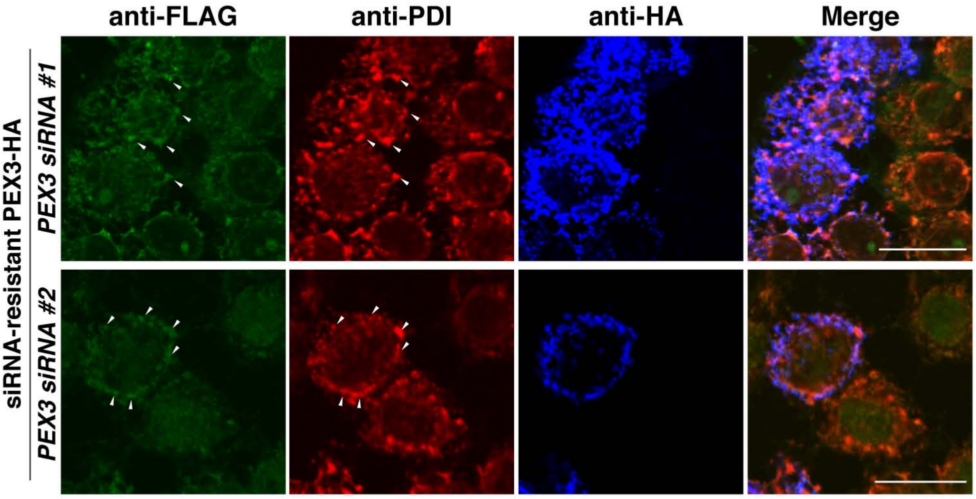

B

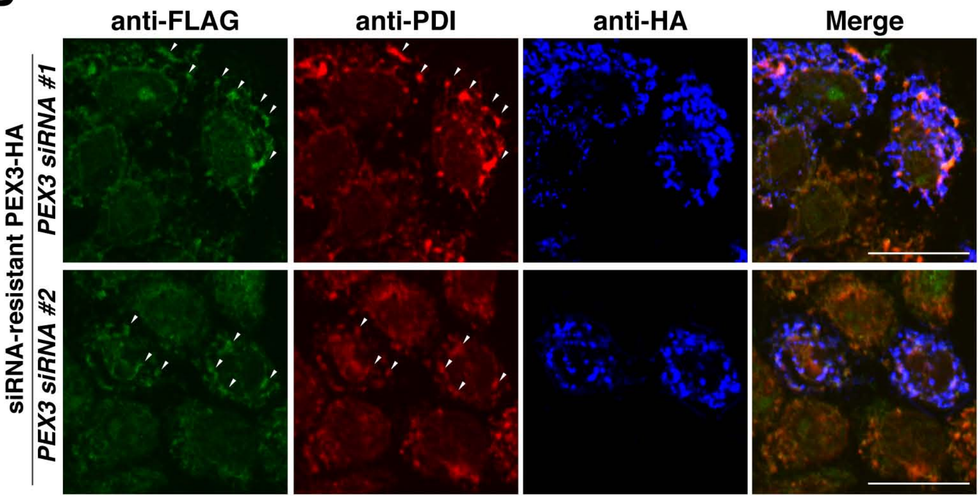

Supplementary Figure 10

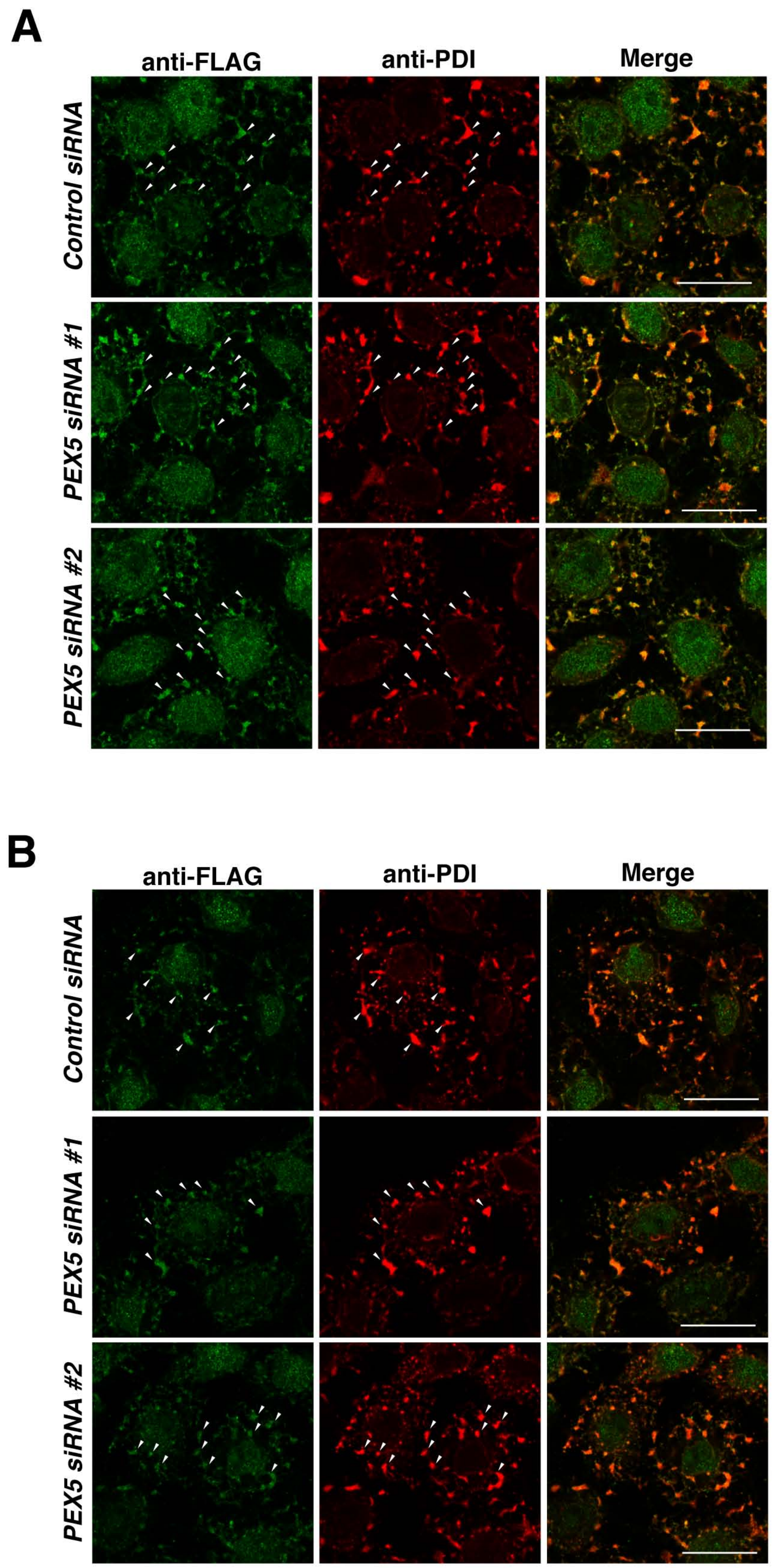

Supplementary Figure 11

Figure 2B

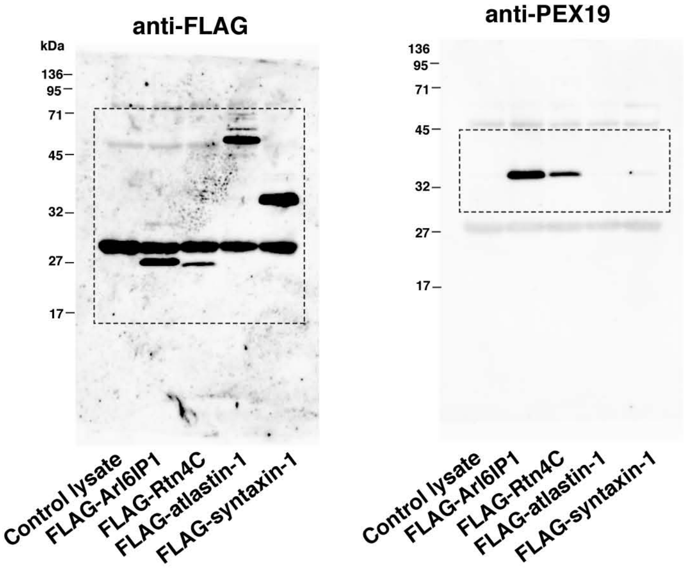

Supplementary Figure 1B

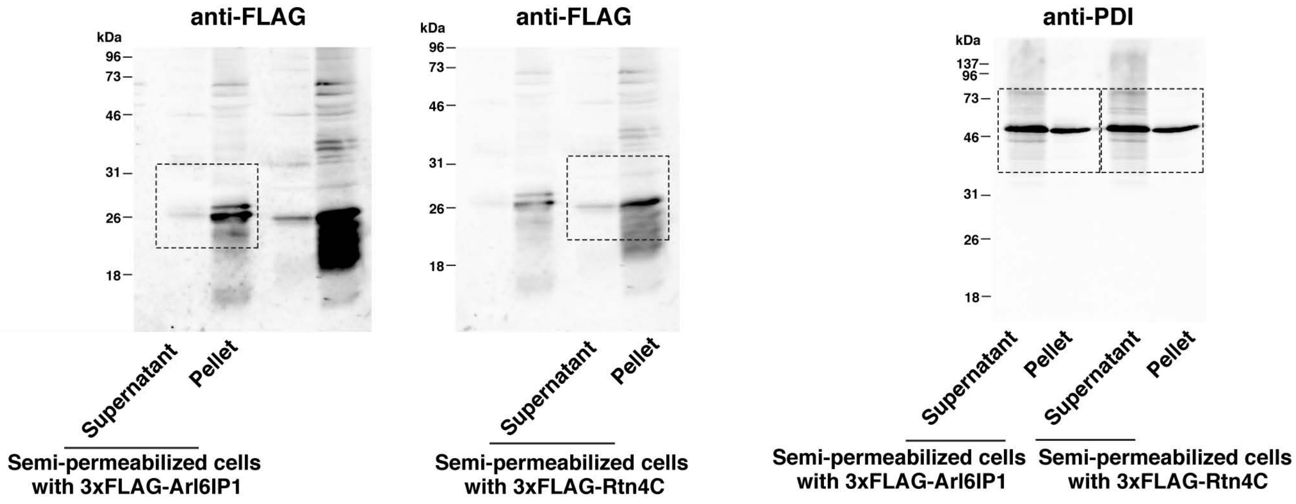

Supplementary Figure 2

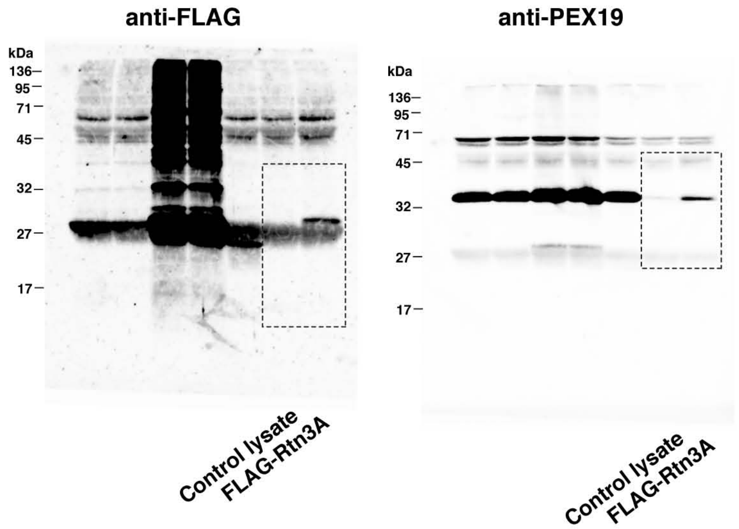

Supplementary Figure 5A

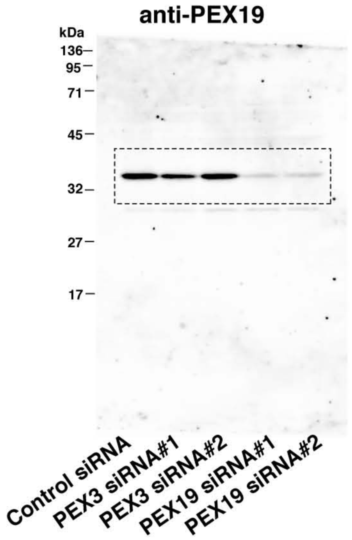

Supplement: Supplementary file 1 — Supplementary figures [file 41598_2018_20797_MOESM1_ESM.pdf]
